# Comparative Analysis of Plasma Extracellular Vesicle Isolation Methods for Purity Assessment and Biomarker Discovery

**DOI:** 10.3390/proteomes13030045

**Published:** 2025-09-18

**Authors:** Alexandra T. Star, Melissa Hewitt, Amanpreet Badhwar, Wen Ding, Tammy-Lynn Tremblay, Jennifer J. Hill, William G. Willmore, Jagdeep K. Sandhu, Arsalan S. Haqqani

**Affiliations:** 1 Human Health Therapeutics Research Centre, National Research Council of Canada, Ottawa, ON K1A 0R6, Canada; alexandra.star@nrc-cnrc.gc.ca (A.T.S.); melissa.hewitt@nrc-cnrc.gc.ca (M.H.); wen.ding@nrc-cnrc.gc.ca (W.D.); tammy-lynn.tremblay@nrc-cnrc.gc.ca (T.-L.T.); jennifer.hill@nrc-cnrc.gc.ca (J.J.H.); jagdeep.sandhu@nrc-cnrc.gc.ca (J.K.S.); 2 Departments of Biology and Chemistry and the Institute of Biochemistry, Carleton University, Ottawa, ON K1S 5B6, Canada; bill.willmore@carleton.ca; 3 Centre de Recherche de l’Institut Universitaire de Gériatrie de Montréal (CRIUGM), Montréal, QC H3W 1W5, Canada; amanpreet.badhwar@umontreal.ca; 4 Ottawa Institute of Systems Biology, Ottawa, ON K1H 8M5, Canada; 5 Department of Biochemistry, Microbiology and Immunology, University of Ottawa, Ottawa, ON K1H 8M5, Canada

**Keywords:** extracellular vesicles, plasma proteomics, purity assessment, enrichment, protein contaminants, biomarker discovery

## Abstract

Background: Extracellular vesicles (EVs) are an important source of blood biomarkers and are emerging as next-generation therapeutics. Demonstrating the purity of isolated EVs is essential for applications ranging from proteomics-based biomarker discovery to biomanufacturing. In this study, we systematically evaluated multiple EV isolation methods for plasma and developed a scoring method to identify the approach best suited for proteomics. Methods: Commonly used enrichment techniques, including size-exclusion chromatography (SEC) and precipitation-based methods, were compared against the starting plasma in terms of particle yield and size, proteomic overlap, depletion of abundant plasma proteins, and enrichment of EV markers and unique proteins. To enable rigorous purity assessment, we established a targeted parallel reaction monitoring (PRM) mass spectrometry assay that quantified key EV markers and contaminant proteins across preparations. Results: Among the methods tested, SEC showed the greatest enrichment of EV markers and unique proteins, with the lowest level of contaminants, resulting in the highest overall purity scores. SEC also allowed for the detection of EV-free proteins. Other methods, by contrast, performed sub-optimally and were less reliable for proteomics-driven biomarker discovery. Conclusions: SEC provides the most EV-enriched plasma isolates for proteomics information, with minimal contamination from plasma proteins. The PRM-based purity scoring offers an objective means of benchmarking EV preparations and may help standardize EV isolation quality for both biomarker discovery and therapeutic manufacturing.

## 1. Introduction

Extracellular vesicles (EVs) are membrane-bound nanoparticles released by cells into the bloodstream, carrying proteins and nucleic acids that reflect the physiological or pathological state of their cells of origin. This rich molecular cargo makes circulating EVs attractive targets for biomarker discovery in diseases such as cancer and metabolic or infectious disorders. However, EV isolation from plasma remains technically challenging. Plasma contains an abundance of proteins and lipoprotein particles that overlap in size or density with EVs, and these contaminants can be co-isolated and interfere with downstream analyses. To date, there is no universally accepted or standardized protocol for purifying plasma-derived EVs, a major limitation in the field. Researchers continue to debate the optimal isolation method, as each approach entails trade-offs in EV yield, purity, and practicality [1,2].

A variety of EV isolation techniques have been employed, each with advantages and limitations. Differential ultracentrifugation (UC) is a classic method that pellets EVs by high-speed centrifugation, but it often co-sediments abundant plasma proteins and other particles alongside EVs. Polymer-based precipitation methods (e.g., polyethylene glycol precipitation kits) can efficiently capture EVs even from small volumes, yet they tend to co-precipitate significant amounts of albumin and other soluble proteins, which diminishes sample purity. Size-exclusion chromatography (SEC) has emerged as a popular alternative for biofluid EV isolation; by passing plasma through a porous resin, EVs can be separated from smaller protein complexes based on size. SEC (often implemented via commercial qEV columns from Izon) yields relatively pure EV fractions with substantially reduced levels of albumin and other contaminants compared to one-step UC or precipitation methods. The main drawback of SEC is that EVs are diluted across eluted fractions, potentially reducing overall yield. Thus, while consensus is growing that SEC provides superior purity for proteomic applications, some studies note that method selection may depend on whether the priority is maximizing EV recovery or maximizing the removal of non-EV proteins.

In recent years, several mass spectrometry (MS)-based studies have directly compared EV isolation methods to determine which best preserves the EV proteome. Notably, Vanderboom et al. (2021) developed an SEC-based workflow (using Izon qEV columns) for plasma EVs and reported that it identified significantly more proteins (with higher quantitative precision) than various conventional isolation techniques [3]. The SEC approach outperformed ultracentrifugation and other single-step methods, establishing SEC as one of the most MS-compatible isolation strategies for plasma EV proteomics [3]. These findings align with other reports that SEC-based isolation, especially using Izon qEV columns, yields deeper proteomic coverage and cleaner EV preparations than precipitation or basic centrifugation methods [1,3]. However, there are also diverging viewpoints. Some investigators continue to utilize one-step UC or precipitation in certain contexts due to higher EV particle yield or convenience, despite the higher co-isolation of contaminants. On the other hand, an emerging strategy is to combine complementary techniques to further enhance EV purity and yield. Hybrid workflows, such as performing ultracentrifugation followed by SEC, have demonstrated improved removal of plasma proteins and increased identification of EV proteins relative to any single method alone. For example, a recent study that incorporated a density cushion ultracentrifugation (DCC) step prior to SEC achieved EV isolates with the highest enrichment of EV exosomal markers and markedly low levels of abundant plasma proteins. Such multi-step approaches can yield a more comprehensive EV proteome at the expense of additional processing time [4,5].

Given the growing recognition that isolation methods profoundly influence downstream EV proteomic profiles, the present study was designed to systematically evaluate multiple EV isolation techniques for plasma and develop a scoring method to identify the approach best suited for proteomics-based biomarker discovery. We compared several commonly used methods for total EV enrichment from plasma, including ExoQuick (EQ), ExoSpin (ES), Izon qEV 35 nm SEC (IZ), Total Exosome Isolation (TI), and High-Select™ Top14 abundant protein depletion kit (T14). Although T14 is not an EV isolation method per se, we included it to test whether depleting abundant plasma proteins would improve EV protein detection. To facilitate objective comparison, we developed a targeted parallel reaction monitoring (PRM) mass spectrometry assay to quantify key EV marker proteins (such as tetraspanins) and contaminant proteins (such as albumin) in each EV preparation. This allowed us to directly measure the purity of EV isolates obtained by each method and identify the best approach for protein biomarker discovery.

## 2. Materials and Methods

### 2.1. Materials

Commercial plasma obtained from BioIVT was thawed on ice. Once thawed, the plasma was centrifuged at 1500× *g* for 10 min to remove any cells or large particles. The supernatant was gently transferred to a new tube and centrifuged again at 10,000× *g* for 10 min to further clear the plasma. These low-speed spins remove residual cells, debris, and large vesicles (e.g., apoptotic bodies or larger microvesicles). Thus, the subsequent isolations are primarily enriching small EVs (exosomes and small microvesicles). We have chosen this standard pre-clearing to focus on the small EV fraction. This pre-clearing is necessary to prevent downstream column clogging (e.g., for SEC) and to improve overall sample consistency per MIBlood-EV recommendations for plasma processing [6]. The supernatant was used for the following isolation methods: Total Exosome Isolation Kit (Invitrogen, Burlington, ON, Canada), ExoQuick Ultra (System Biosciences, Palo Alto, CA, USA), ExoSpin (Cell Guidance Systems, St. Louis, MO, USA), High-Select™ Top14 abundant protein depletion (ThermoFisher Scientific, San Jose, CA, USA), and qEV original 35 nM (Izon Science, Medford, MA, USA). The Total Exosome Isolation Kit required 500 µL of plasma, while ExoQuick and ExoSpin required 250 µL of plasma, and the qEV columns required 150 µL of plasma. The ExoQuick and qEV sample volumes were reduced by about half by Speedvac (Labconco, Kansas, MO, USA) for 2 h to further concentrate the samples. The protein concentration of each sample was determined by Bradford Assay (BioRad, Mississauga, ON, Canada). The samples were frozen and stored at −80 °C for 48 h between EV isolation and sample digestion by SP3 method. All experiments were conducted with a minimum of three independent replicates and, where possible, adhered to the updated MISEV 2023 guidelines for blood EV research [7], ensuring rigorous and standardized reporting of sample handling, EV isolation, and characterization.

### 2.2. Total Exosome Isolation Kit

The Total Exosome Isolation Kit (ThermoFisher Scientific) was used according to the manufacturer’s instructions. Here, 100 µL of the Total Exosome Isolation Reagent was added to 500 µL of plasma on ice. This solution was vortexed and then returned to ice for 30 min. Once the incubation was complete the sample was centrifuged at 10,000× *g* for 15 min. The supernatant was transferred to a new tube and used for the protein assay. The pellet was re-suspended in 100 µL of 1× PBS.

### 2.3. ExoQuick Ultra

The ExoQuick Ultra kit was used according to the manufacturer’s instructions. Here, 67 µL of the ExoQuick Exosome Precipitation Solution (System Biosciences) was added to 250 µL of plasma, mixed by inverting the tube, and incubated on ice for 30 min. The sample was centrifuged at 3000× *g* for 10 min. The supernatant was discarded, and the pellet was re-suspended in 200 µL of Buffer B followed by 200 µL of Buffer A and then added to the purification columns. The sample was incubated in the purification column for 5 min at room temperature and then eluted with 100 µL Buffer B and 400 µL of sample Buffers A and B. The sample was concentrated (half its volume) with a Speedvac (2 h).

### 2.4. ExoSpin

The ExoSpin Kit was used according to the manufacturer’s instructions. First, 125 µL of ExoSpin Buffer was added to 250 µL of plasma and mixed by inverting the tube. The mixture was incubated on ice for one hour. The mixture was centrifuged at 16,000× *g* for 30 min. The supernatant was carefully removed and discarded, and the pellet was re-suspended in 100 µL of 1× PBS. The re-suspended pellet was carefully applied to the purification column. Three 200 µL elutions of 1× PBS were collected from the column.

### 2.5. Izon SEC

The qEVsingle 35 nm column (Izon Science) was used according to the manufacturer’s instructions. The column was equilibrated at room temperature and with 1× PBS by loading two column volumes worth prior to the addition of 150 µL of plasma. Successive additions of 200 µL of PBS were added to elute each fraction from the column. Fractions 6, 7, and 8 were pooled, concentrated with a Speedvac, and used for comparison with other isolation methods.

### 2.6. Nanoparticle Tracking Analysis ZetaView

Plasma EV samples obtained from the above-mentioned kits were appropriately diluted and analyzed for nanoparticle content using a ZetaView PMX-420 Nanoparticle Tracking Analysis (NTA) system (Particle Metrix, Inning am Ammersee, Germany). The instrument was calibrated with commercially available 100 nm polystyrene beads (Particle Metrix, cat. no. 110-0020), diluted 1:250,000, to ensure accurate measurements of particle size and concentration. Samples were diluted in 0.1 µm filtered PBS, degassed using water-bath sonication, to achieve a particle count of 40–200 particles per frame. Once the sensitivity, particle/frame count, and particle drift were within the acceptable range, measurements in scatter mode were performed by scanning 11 cell positions and capturing 30 frames per position using the following settings: camera sensitivity, 85; shutter speed, 40; focus, autofocus; scattering intensity, automatic; minimum brightness, 15; minimum size (pixels), 10; maximum size (pixels), 500; and cell temperature, 25 °C. Each sample was measured in triplicate. Data were acquired using the instrument’s built-in software (version 8.05.11 SP4) with a particle bin size of 5 nm and a minimum path length of 5. The resulting data were extracted from the output files and plotted using GraphPad Prism software version 10.5.0 (774).

### 2.7. Protein Processing for Mass Spectrometry Analysis

Concentrations of the EV preparations from the various methods were determined by Bradford Assay (BioRad) following the manufacturer’s instructions. For shotgun proteomics, the EVs were lysed by the addition of 10% SDS (BioRad) for a final concentration of 1% in the sample. The samples were reduced by adding dithiothreitol (DTT, Sigma-Aldrich, Oakville, ON, Canada) at a final concentration of 5 mM and incubating at 80 °C for 10 min. The samples were alkylated with 2-iodoacetamide (IAA, Sigma-Aldrich) at a final concentration of 10 mM and incubated at room temperature in the dark for 23 min. The IAA was then quenched with another addition of 5 mM DTT for 12 min and room temperature. Protein digestion was performed following the SP3 protocol from Hughes et al., 2019 [8]. Briefly, a combination of two SpeedBead Magnetic Carboxylate Modified Particles (Sigma-Aldrich) was added at 1:1. The beads were added in 10× excess of the protein present in the samples. Protein binding to the beads was achieved by adding them 1:1 with ethanol (Commercial Alcohols, Toronto, ON, Canada) and incubating them for 15 min at room temperature, with mixing up and down with pipette every 5 min. The supernatant was removed, and the beads were subsequently washed three times with 80% ethanol for 10 min. After removal of the last wash, the samples and beads were re-suspended in a volume of 0.008 mg/mL Sequencing-Grade Modified Trypsin solution (Promega, Madison, WI, USA) diluted with 50 mM ammonium bicarbonate (Sigma-Aldrich) to yield a protein concentration of 0.1 mg/mL for each sample. The samples were incubated at 37 °C overnight. The next day, the samples were acidified with formic acid to obtain a final concentration of 0.1% formic acid in the sample. Equal protein for all samples (0.1 µg) was loaded onto the LC column for nLC-PRM or nLC-MS/MS analysis.

### 2.8. Liquid Chromatography Tandem Mass Spectrometry (LC-MS/M) and PRM Analysis

Samples were analyzed by label-free mass spectrometry-based quantification and PRM. For proteomics analysis of the generated peptides, 0.1 µg of digested sample was analyzed by nano-LC-MS/MS or by nano-LC-PRM on a Thermo Scientific Orbitrap Eclipse™ Tribrid™ MS (ThermoFisher Scientific) coupled with an UltiMate™ 3000 RSLCnano System with Dionex ProFlow Meter (ThermoFisher Scientific). Briefly, 10 µL of peptide solution was concentrated on a PEPMAP NEO C18 5 µm trap (300 µm × 5 mm, Thermo Scientific) and then subsequently separated on a nanoAcquity UPLC M-Class 1.7 um BEH C18 column (100 µm × 100 mm), 130 Å pore size (Waters, Milford, MA, USA), using a flow rate of 500 nL/min with a 72 min step-wise gradient of 1% to 6% Solvent B (Solvent A: 0.1% formic acid; Solvent B: 100% acetonitrile (ACN)/0.1% formic acid) for 4 min, followed by a 48 min ramp to 25% Solvent B, a 9 min ramp to 40% Solvent B, another 3 min ramp to 85% Solvent B, and an 8 min equilibration at 1% Solvent B. Blanks with a 30 min gradient were run between samples to clean the system and reduce carry-over between runs. A full MS scan was acquired in the Orbitrap between 350 and 1800 *m*/*z* in profile mode at 60,000 resolution and was followed by a data-dependent MS/MS scan in the ion trap (IT) after higher-energy collisional dissociation (HCD) activation. Ions were excluded after a repeat count of 1 for a duration of 60 s. All data were recorded with Xcalibur software version 4.4.16.14 (build 6 February 2020) (ThermoFisher Scientific). All PRM spectra were acquired in the Orbitrap mass analyzer at a resolving power of at least 30,000 (at *m*/*z* 400). Peptide targets were selected from prior DDA experiments and consisted of high-confidence proteotypic peptides. A scheduled inclusion list was used to maximize sampling efficiency: each acquisition cycle consisted of one full MS1 survey scan followed by up to 30 targeted MS2 (PRM) events. MS1 scans were acquired across *m*/*z* 350–1800 (resolution 60,000). MS2 product ion spectra were acquired in the Orbitrap at 30,000 resolution (AGC 100%, max IT 50 ms). Targeted precursors were isolated and fragmented by HCD. Raw PRM data were processed using targeted extraction of the top product ions per precursor using Skyline version 24.1.0.199 (6a0775ef83), with manual review of peak integration and retention time alignment.

### 2.9. Data Analysis

Thermo raw files from DDA runs were processed with FragPipe version 23.0. The built-in FragPipe “LFQ-MBR” workflow was used. Briefly, MSFragger (v4.3) was used to perform a closed search followed by label-free quantification with match-between-runs enabled using IonQuant (v1.11.11). In the closed search, both the initial precursor and fragment mass tolerances were set to 20 ppm. The enzyme was set to strict trypsin, and the maximum allowed missed cleavage was set to 2. Methionine oxidation and protein N-terminal acetylation were set as variable modifications, and carbamidomethylation of cysteine was set as a fixed variable modification. The database used was the human Uniprot database (2025_03, 20,421 entries) with decoys added. Peptide-spectrum matches were filtered to a 1% false discovery rate (FDR) at the peptide and protein levels using Philosopher v5.1.1. Most of the peptide results were imported into Skyline version 24.1.0.199 (6a0775ef83), and peaks were manually validated for identification and quantification. For PRM analysis, raw files were imported into Skyline for chromatographic peak integration and quantification. For statistical analysis, GraphPad Prism version 10.5.0 (774) was used.

### 2.10. Calculation for Purity Assessment

The scoring to rank the different methods for the ability to discover proteomics biomarkers was based on two key criteria: the depletion of contaminating abundant proteins and enrichment of EV markers. For the first criterion, to evaluate how well each EV isolation method removes abundant non-EV contaminants, we adapted the contamination index method from Okaty et al. (2011) [9]. Originally developed for transcriptomic data, this index is defined as the average normalized level of off-target “negative” markers in a sample (e.g., glial cell gene markers in what should be a pure neuron sample). A higher contamination index ($S^{-}$) indicates a greater presence of these off-target signals (lower purity), whereas a lower value implies that contaminating components (e.g., abundant plasma proteins in the EV context) have been effectively depleted, yielding a purer EV preparation. The second criterion uses a projection-based enrichment score inspired by Sugino et al. (2019) [10], which quantifies the enrichment of positive markers in each sample. The enrichment score ($S^{+}$) is defined as the fraction of the total protein signal attributed to the pure EV reference (i.e., the NNLS coefficient for the EV component divided by the sum of all coefficients for that sample). Higher enrichment scores indicate samples whose composition is dominated by EV-specific proteins, whereas lower scores suggest poor enrichment. Since a correlation was seen between the contamination index and the enrichment score, to capture both metrics, we calculated a single composite score for EV purity based on Technique for Order of Preference by Similarity to Ideal Solution (TOPSIS) [11]. In TOPSIS, each method is evaluated by its Euclidean distance from both an ideal solution (maximum enrichment and minimum contamination) and an anti-ideal solution (minimum enrichment and maximum contamination). The composite score $T_{i}$ for method *i* is then calculated as the ratio of a method’s distance from the anti-ideal over the sum of its distances from both ideal and anti-ideal:

(1) $$T_{i}=\frac{E_{i}^{-}}{\left(E_{i}^{-}+E_{i}^{+}\right)}\;,$$

where $E_{i}^{+}$ is the Euclidean distance of method *i* from the positive control (EV), and $E_{i}^{-}$ is the Euclidean distance of the method from the negative control (PL). Methods with $T_{i}$ values closer to 1 are considered better, as they lie nearer the ideal EV state and further from the plasma baseline, whereas $T_{i}$ values near 0 denote poor performance. This approach of multi-criteria ranking thus integrates both depletion and enrichment metrics into a single, objective score and was used for comparing EV isolation methods.

## 3. Results

### 3.1. Number of Particles

**Figure 1:**
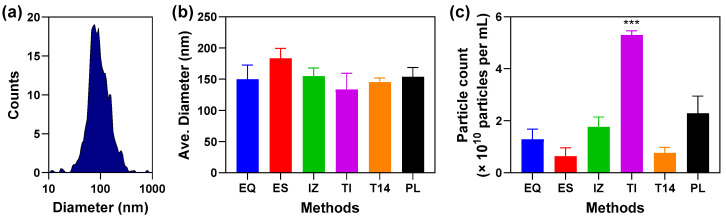
Nanoparticle tracking analysis of EVs isolated from plasma using five different methods. (**a**) Representative particle size distribution from an EQ preparation showing counts versus diameter. (**b**) Mean particle diameter (nm) for each method (mean ± SD, *n* ≥ 3 independent preparations). No statistically significant differences were observed among methods by one-way ANOVA. (**c**) Particle yield is expressed as the number of particles isolated per mL of starting plasma (×10^10^ particles per mL; mean ± SD, *n* ≥ 3 independent preparations). Data were analyzed using one-way ANOVA followed by Tukey’s post hoc test, and statistical significance is shown as *** *p* < 0.001, indicating that TI yielded significantly more particles than all other methods. EQ = ExoQuick, ES = ExoSpin, IZ = Izon qEV-35 nm column, TI = Total Exosome Isolation, T14 = Top14 abundant protein depletion kit, and PL = starting plasma.

Various methods for enriching total, small EVs from plasma were compared, including ExoQuick (EQ), ExoSpin (ES), Izon qEV 35 nm SEC (IZ), Total Exosome Isolation (TI), and the High-Select™ Top14 abundant protein depletion kit (T14). To adhere to MISEV2023 recommendations for minimal information for studies of EVs [7], nanoparticle tracking analysis using a ZetaView instrument was employed to assess particle concentration and size for each preparation (Figure 1). Because each method requires a different starting plasma volume and results in varying final EV preparation volumes, particle count was normalized to the number of particles per milliliter of plasma (Supplementary Table S1). Most methods yielded comparable particle counts, averaging between 6 and 20 billion isolated particles per mL of plasma. However, the TI method produced the highest yield, with over 50 billion particles per mL (Figure 1c), a statistically significantly increase of at least 2-fold compared to the other methods. No statistically significant differences in particle size distribution were observed, with all methods isolating particles of about 140 nm (range 100–200 nm) (Figure 1b). Taken together, these results show that TI method provides the highest particle yield, though additional quality metrics are necessary to fully evaluate and compare the performance of each EV isolation method.

### 3.2. Proteome Overlap

**Figure 2:**
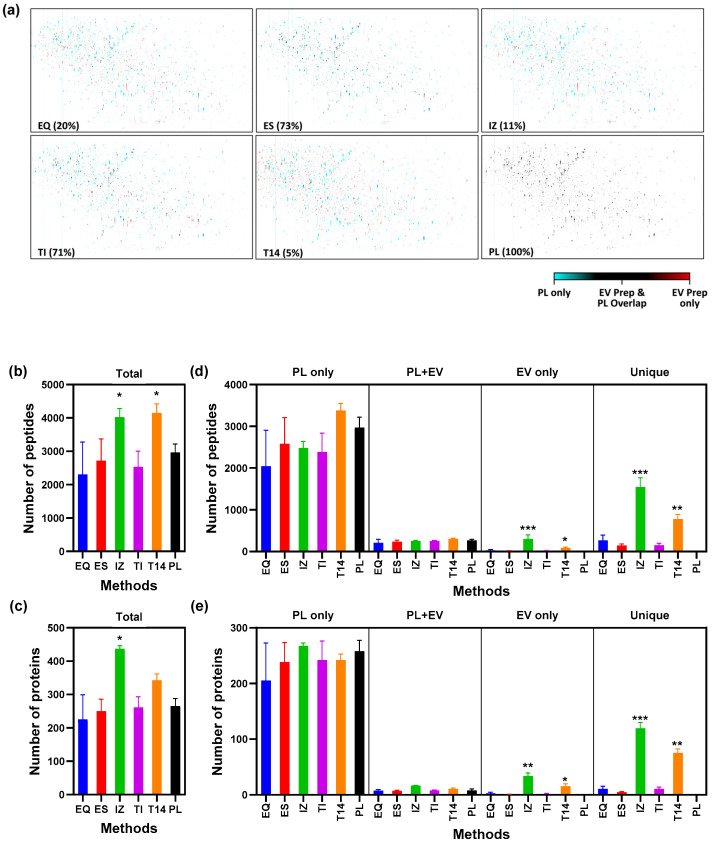
Comparative proteomic characterization of EV isolates vs. starting plasma. (**a**) MS1 image from each EV preparation was overlayed on image from PL (starting plasma) to identify shared features. Each spot on the image represents a peptide ion and has a cyan, red, or black color. The cyan color indicates the ion is predominantly present in the EV prep method, and the red color indicates the ion is predominantly present in the starting plasma PL, while the black color indicates the ion is common/overlaps between the EV prep method and PL. The numbers in parentheses indicate the percentage overlap with PL. (**b**,**c**) Total number of peptides (**b**) and their corresponding proteins (**c**) identified by each method, mean ± SD (*n* ≥ 3 independent preparations). (**d**,**e**) Breakdown of peptides (**d**) and proteins (**e**) identified by each method into categories: “PL only” (detected in starting plasma only), “EV-only markers” (canonical EV marker proteins found only in EV isolates, not in plasma), “PL + EV markers” (canonical EV markers found in both plasma and EV isolates), and “Unique” (other proteins detected only in EV isolates and absent in plasma). Asterisks represent statistically significant differences (Kruskal–Wallis test, Dunn’s multiple-comparisons test, and adjusted *p*-values): * *p* < 0.05; ** *p* < 0.01; *** *p* < 0.001. EQ = ExoQuick, ES = ExoSpin, IZ = Izon qEV-35 nm column, TI = Total Exosome Isolation, T14 = Top14 abundant protein depletion kit, and PL = starting plasma.

Proteins were extracted from each total EV preparation (EQ, ES, IZ, TI, and T14), digested using the SP3 method, and analyzed by bottom-up proteomics (nano-LC-MS/MS DDA), and their proteomes were compared with that of the starting plasma (PL). An equal protein amount (0.1 µg) was loaded for each nano-LC-MS/MS analysis, despite the fact that the protein concentrations of each EV isolate varied among the methods (Supplementary Table S1), likely reflecting the variability of presence of contaminating proteins. To compare the proteome overlap, MS1 images from each EV isolate were overlayed on MS1 images of PL to identify shared features (MSight images, Figure 2a). The fraction of peaks overlapping with PL was 20% for EQ, 73% for ES, 11% for IZ, 71% for TI, and 5% for T14, with the shared signals predominantly corresponding to high-abundance plasma proteins (which was confirmed below by relative LFQ intensity comparison). While image overlaying is a coarse measure to compare proteome and more detailed protein-level comparison is provided below, these findings nonetheless indicate that IZ, EQ, and T14 isolations yield EV proteomes that are markedly distinct from the original plasma, which may reflect enrichment of EV-specific proteins, whereas ES and TI preparations retain proteomes highly similar to PL, suggesting substantial plasma protein retention.

The number of peptides and corresponding proteins identified by DDA analysis of each prep are shown in Figure 2b–e. A total of about 2700 peptides, corresponding to about 240 proteins, were identified in PL (Figure 2b,c). Only IZ and T14 showed an increased number of peptides and proteins relative to PL, whereas the other methods did not show a difference compared to PL. The difference was statistically significant for IZ at both the peptide and protein levels, while for T14, only peptides showed a statistically significant difference (Figure 2b,c). The identified proteins were classified as PL only, EV only markers, PL + EV markers, or unique to EV preps, as defined in Figure 2d,e. For all EV preparations, no significant reduction compared to the PL proteome in the number of peptides and proteins was seen for the PL-only panel (Figure 2d,e), suggesting that while the overall overlap might be different (i.e., depleted, Figure 2a), the number of proteins appear to be the same. IZ and T14 showed a significant increase in the number of peptides corresponding to proteins that are EV markers and those that are unique to the EV preparations (i.e., absent in PL) (Figure 2d,e).

### 3.3. Contaminating Abundant Plasma Proteins

**Figure 3:**
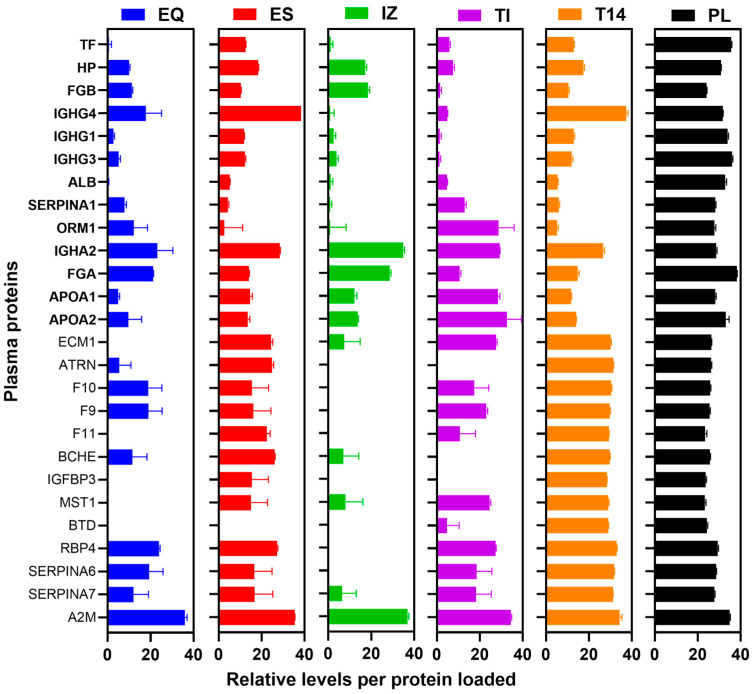
Relative levels of 26 abundant plasma proteins (including albumin and immunoglobulins) in the 5 EV preparations (EQ, ES, IZ, TI, and T14) and starting plasma PL. Tryptic digests (0.1 µg protein) of each EV preparation and starting PL were loaded onto nano-LC-MS/MS and analyzed by DDA. The peptide LFQ intensities for each indicated protein are plotted as log2 ± SD (*n* ≥ 3 independent preparations) following mean normalization. Most of the proteins showed depletion depending on the EV preparation. Proteins in bold (from TF to APOA2) are supposed to be depleted by the T14 kit. EQ = ExoQuick, ES = ExoSpin, IZ = Izon qEV-35 nm column, TI = Total Exosome Isolation, T14 = Top14 abundant protein depletion kit, and PL = starting plasma.

The evaluation of plasma EV biomarkers is often hampered by highly abundant plasma proteins [4], which consume instrument sequencing capacity, mask lower-abundance species, and limit how much sample can be loaded onto a nano-LC-MS system. Effective depletion of these abundant proteins is therefore essential for achieving deeper proteome coverage in EV samples. To evaluate these “contaminants”, proteins detectable in whole plasma (PL) were compared to those in each EV isolation to assess the removal of high-abundance species. The quantitative comparison (based on LFQ comparison) of the top 26 most abundant plasma contaminants is shown in Figure 3, while the remaining plasma-specific proteins are presented in Supplementary Table S2. All EV isolation methods tested (IZ, EQ, ES, and TI) removed some of the highly abundant plasma proteins such as albumin, transferrin (TF), and α-1-acid glycoprotein (ORM1), although each method differed in which specific proteins were most effectively depleted. All methods also reduced the levels of several medium-abundance proteins (e.g., DEFA1, APOF, IGF2, VASN, S100A9, PI16, and LRG1), which are typically in the 1–100 µg/mL range in plasma (mid-tier abundance as per HUPO Plasma Proteome dataset [12]). The data for these proteins are included in our panel (Figure 3 and Supplementary Table S2), showing partial depletion alongside the high-abundance targets. The Top14 (T14) abundant protein depletion kit removed the majority of its 14 target proteins (which together constitute ~95% of total plasma protein content), though a few targets like apolipoprotein A-I/A-II were still detected. This kit also removed some proteins it was not designed to, such as hemoglobin subunit beta (HBB), likely due to non-specific interactions. Overall, IZ and T14 were most effective in depleting the abundant plasma proteins, whereas other kits reduced them but were not as effective.

### 3.4. EV-Specific Markers

**Figure 4:**
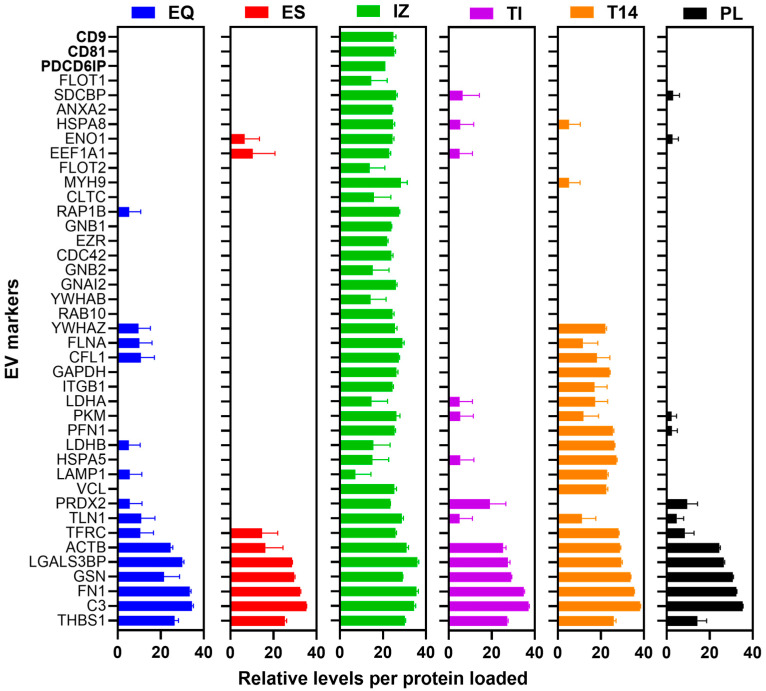
Relative levels of 41 of the most frequently observed EV markers detected in the 5 EV preparations (EQ, ES, IZ, TI, and T14) and starting plasma PL. Tryptic digests (0.1 µg protein) of each EV preparation and starting PL were loaded onto nano-LC-MS/MS and analyzed by DDA. The peptide LFQ intensities for each indicated protein are plotted as log2 ± SD (*n* ≥ 3 independent preparations) following mean normalization. Tetraspanins are shown in bold. EQ = ExoQuick, ES = ExoSpin, IZ = Izon qEV-35 nm column, TI = Total Exosome Isolation, T14 = Top14 abundant protein depletion kit, and PL = starting plasma.

We next examined whether known EV-specific proteins (markers) were detectable in the proteomes of each isolation method. Using a compiled list of the top 100 proteins most frequently identified in EVs from the ExoCarta [13] and Vesiclepedia [14] databases (with generic plasma proteins like albumin and A2M excluded), we counted how many of these putative EV markers were found in each EV isolate (Figure 4 and Supplemental Table S3). In total, 68 of the 100 marker proteins were detected across all samples. The IZ (size-exclusion) isolates contained the most (62 out of 100), markedly more than any other method (T14: 35; EQ: 17; TI: 15; ES: 10). For example, well-known exosomal markers such as the tetraspanins CD81 and CD9, as well as Alix (PDCD6IP), flotillin-1 (FLOT1), syntenin-1 (SDCBP), annexin A2 (ANXA2), elongation factor 1-alpha (EEF1A1), enolase 1 (ENO1), and the heat shock cognate HSPA8, were only detected in the IZ isolates. Likewise, certain EV-associated proteins, including CD44, glucose-6-phosphate isomerase (GPI), SLC3A2 (CD98 heavy chain), and annexins A5 (ANXA5) and A1 (ANXA1), were found exclusively in the T14 isolates. In contrast, some proteins identified as “EV markers” in the EQ, ES, and TI preparations were also present in the starting plasma (e.g., ACTB, LGALS3BP, GSN, FN1, C3, TLN1, and THBS1), suggesting they were co-isolated plasma components rather than true EV-enriched proteins. Overall, the IZ method captured by far the broadest array of EV-specific marker proteins, highlighting its superior performance in isolating EV components compared to the other methods.

### 3.5. Unique Proteins

**Figure 5:**
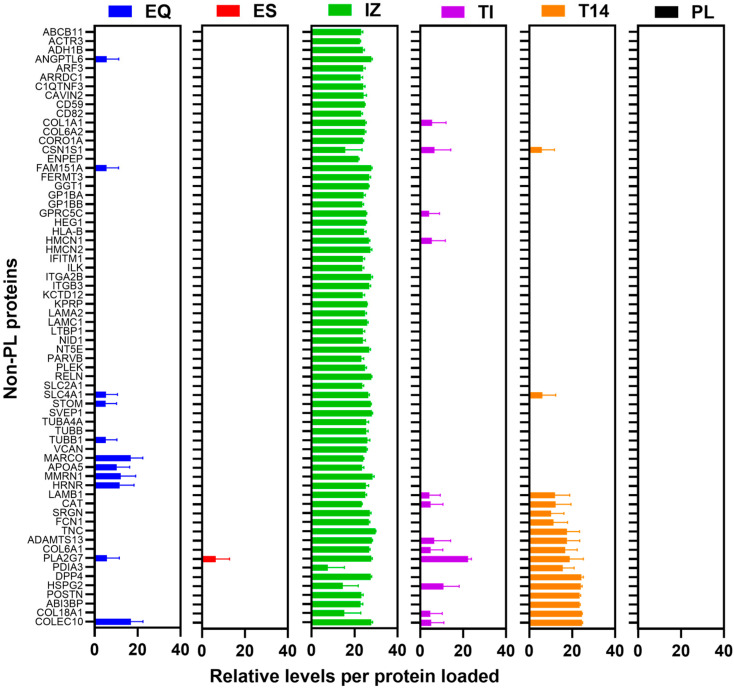
Relative levels of 66 unique proteins detected in the 5 EV preparations (EQ, ES, IZ, TI, and T14) but absent in the starting plasma PL. Tryptic digests (0.1 µg protein) of each EV preparation and starting PL were loaded onto nano-LC-MS/MS and analyzed by DDA. The peptide LFQ intensities for each indicated protein are plotted as log2 ± SD (*n* ≥ 3 independent preparations) following mean normalization. EQ = ExoQuick, ES = ExoSpin, IZ = Izon qEV-35 nm column, TI = Total Exosome Isolation, T14 = Top14 abundant protein depletion kit, and PL = starting plasma.

An important advantage of EV isolation is the ability to detect novel proteins not observable in whole plasma, since such proteins could serve as new biomarkers. We therefore identified proteins that were not detected in the starting plasma but appeared in the EV isolates (Figure 5). Over 150 such proteins were found in total (Supplemental Table S4), consistent with reports that removing abundant plasma proteins can unveil on the order of hundreds of additional low-abundance proteins. Notably, the vast majority of these unique proteins came from either the IZ method (61 proteins unique to IZ) or the T14 method (54 unique to T14), with an overlap of 12 proteins found only in those two. In contrast, each of the other isolation methods (EQ, ES, and TI) yielded fewer than five proteins that were not already present in plasma. Thus, the IZ and T14 approaches were far more effective in revealing proteins absent in whole plasma, underscoring their benefit for discovering novel EV-associated biomarkers.

### 3.6. PRM Analysis

**Figure 6:**
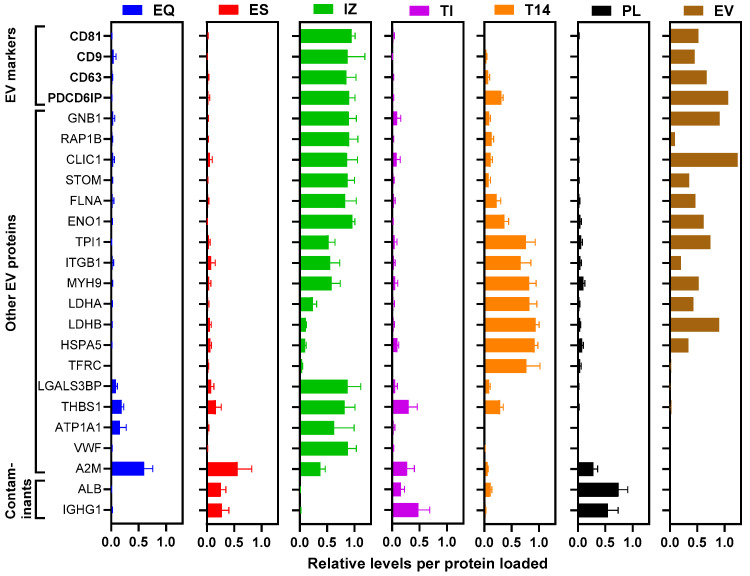
PRM analysis of positive and negative markers in 5 EV preparations (EQ, ES, IZ, TI, and T14), starting plasma PL, and positive control EV. Tryptic digests (0.1 µg protein) of each EV preparation and starting PL were analyzed by PRM for 50 peptides corresponding to the shown 24 proteins. The summed peptide intensities for each indicated proteins were mean normalized amongst the samples and plotted as the mean ± SD (*n* ≥ 3 independent preps). Tetraspanins are shown in bold. EQ = ExoQuick, ES = ExoSpin, IZ = Izon qEV-35 nm column, TI = Total Exosome Isolation, T14 = Top14 abundant protein depletion kit, PL = starting plasma, and EV = EVs from A549 cells.

As label-free DDA can be biased towards high-abundance proteins resulting in under-sampling and limited quantitative accuracy, we next used a targeted PRM MS/MS approach focusing on key EV markers and common contaminants to compare the isolation methods. To ensure we adhere to MISEV recommendations, we developed a PRM assay monitoring 50 peptide ions corresponding to known EV-enriched proteins (e.g., tetraspanins CD9, CD63, CD81; ALIX; TSG101; Annexin A2; Flotillin-1; HSP70 family) and typical plasma contaminants (e.g., albumin and immunoglobulins) (Figure 6 and Supplemental Table S5). This panel was chosen to quantitatively represent positive EV markers and negative markers of purity (Supplemental Table S6). A method with few candidate biomarker peptides was also used. Consistent with the DDA findings, the PRM analysis showed that the contaminant proteins were strongly depleted in the IZ, EQ, and T14 isolates: peptides from abundant plasma proteins (albumin and IgG) were reduced by >90% in those preparations, whereas depletion was less pronounced for ES and TI (approximately 60–75% removal). Likewise, peptides specific to bona fide EV markers produced high signals in the IZ-derived EV samples but were much lower in abundance in the EV preparations from EQ, ES, TI, and T14. Notably, the IZ isolates also yielded the greatest number of detectable EV-associated proteins and lowest contaminant signals of all methods in the PRM assay, significantly more than any other method, mirroring the trend observed in the untargeted proteomics. These targeted data quantitatively reinforce that the IZ (SEC) method produces the cleanest and most EV-enriched preparations, outperforming the other isolation techniques in purifying EVs while removing contaminants.

### 3.7. Purity Assessment

**Figure 7:**
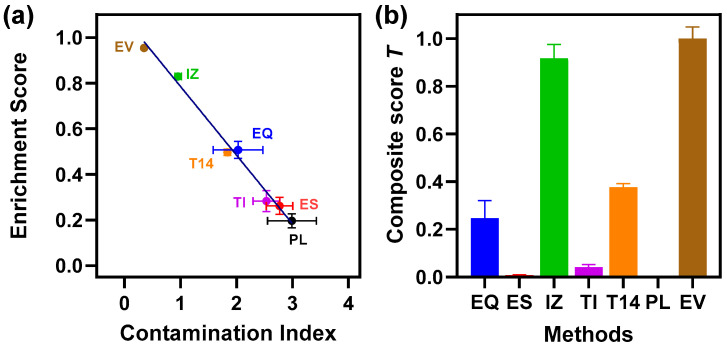
Assessment of EV enrichment for 5 EV preparations (EQ, ES, IZ, TI, and T14) relative to starting plasma PL and positive control EV. Relative levels of positive and negative EV markers (e.g., tetraspanins and albumin) from PRM results were used to calculate the (**a**) EV enrichment score and abundant protein contamination index, which were then used to calculate (**b**) the final composite score to rank the best EV isolation method for biomarker discovery. Results are plotted as the mean ± SD (*n* ≥ 3 independent preps). EQ = ExoQuick (blue symbol and bar), ES = ExoSpin (red symbol and bar), IZ = Izon qEV-35 nm column (green symbol and bar), TI = Total Exosome Isolation (purple symbol and bar), T14 = Top14 abundant protein depletion kit (orange symbol and bar), PL = starting plasma (black symbol and bar), and EV = EVs from A549 cells (brown symbol and bar).

The PRM results allowed us to evaluate EV sample purity using defined quantitative metrics. From the PRM results, we computed two metrics for each sample: a “contamination index”, defined as the mean-normalized abundance of plasma-derived “negative” marker peptides (albumin, immunoglobulins, etc.), and an “enrichment score”, defined as the fraction of total PRM signal coming from EV-specific “positive” marker peptides (e.g., tetraspanins). The specific markers used for the metrics are listed in Supplementary Table S6. As shown in Figure 7a, these two metrics were inversely correlated (methods with low contamination naturally had high EV enrichment). For benchmarking, EVs isolated from a cultured cell line (A549) were used as a positive high-purity control, whereas whole plasma served as a negative control (representing maximal contamination). The purity metrics clearly reflected the differences between methods. As expected, the EVs from the cell line (positive control) showed a very low contamination index (~0.35) and a high enrichment score (~0.954), while unprocessed plasma (negative control) had the worst values (contamination index ~3.0; enrichment ~0.2). Among the plasma isolation methods, the IZ method achieved by far the best purity: its contamination index (~1.0) was the lowest, and its enrichment score (~0.83) the highest, approaching the purity of the cell-derived EVs. The next best method was the T14 depletion approach (contamination ~1.8; enrichment ~0.50), followed by EQ (contamination ~2.0; enrichment ~0.51). In contrast, the EV preparations obtained via TI and ES had substantially higher contamination indices (~2.5 and ~2.8, respectively) and very low enrichment scores (0.28 for TI and 0.26 for ES). We further combined the two complementary scores into a single composite purity score using a TOPSIS multi-criteria algorithm [11], which ranks each method from 0 to 1. The composite scores (Figure 7b) reflected these quantitative indices, underscoring that the IZ (SEC) method yields the “cleanest” EV isolate from plasma, with markedly less non-EV carryover and higher relative EV content than any of the other techniques. Implementing such purity scoring provides an objective means to compare and optimize EV isolation protocols moving forward.

### 3.8. SEC Fractions

**Figure 8:**
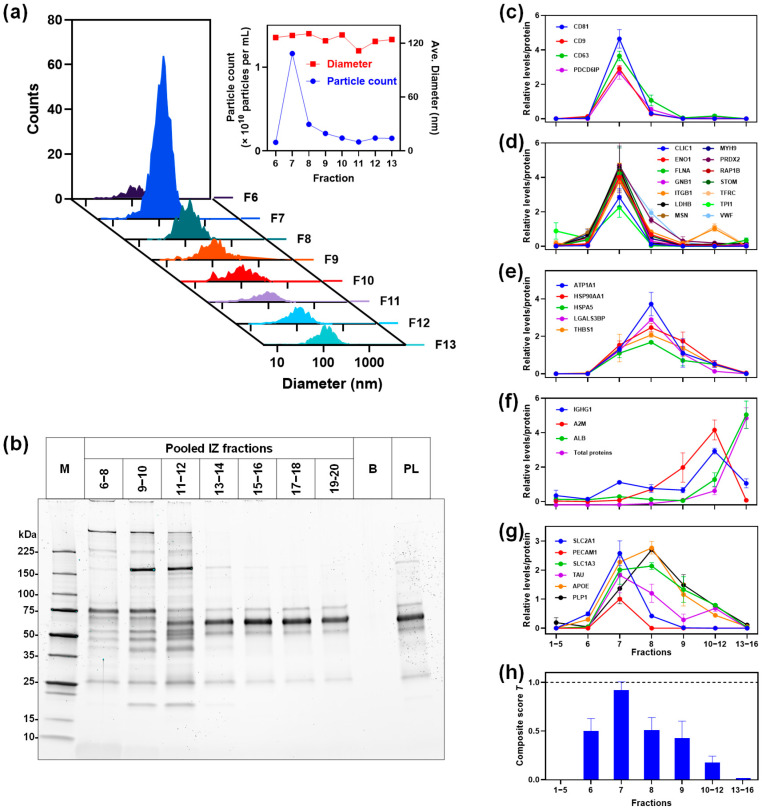
Analyses of IZ fractions. (**a**) Nanoparticle tracking analysis of fractions 6 to 13 showing particle size distribution (counts versus diameter). Inset shows the number of particles isolated per mL of starting plasma (×10^10^ particles per mL on left axis) and mean particle diameter (nm, on right axis) for each fraction. (**b**) Coomassie blue-stained SDS-PAGE of pooled IZ fractions and starting plasma. Lanes: protein molecular weight markers (M), pooled IZ fractions 6–8, 9–10, 11–12, 13–14, 15–16, 17–18, and 19–20, blank lane (B), and starting plasma (PL). The prominent protein at around 65 kDa in PL and fractions 13 and higher corresponds to albumin. (**c**–**g**) PRM analysis of IZ fractions for tetraspanins (**c**), positive EV markers co-eluting with tetraspanins (**d**), positive EV markers co-eluting with tetraspanins (**e**), negative markers (contaminating proteins) (**f**), biomarkers (**g**), and composite scores for EV enrichment (**h**).

As SEC-based methods allow for fractionation of plasma components, we further explored whether collecting sequential SEC fractions could uncover additional unique proteins. We collected sequential fractions from the Izon qEV SEC column and analyzed them by targeted PRM assays (Figure 8), as described above. NTA revealed a sharp peak in particle concentration in fractions 6–8 (~1.8 × 10^10^ particles/mL, mean diameter ~140 nm), with counts falling rapidly after fraction 9 (Figure 8a). SDS-PAGE showed that these early pools were largely devoid of the albumin and immunoglobulin bands that dominated later fractions (Figure 8b). PRM analysis revealed that the canonical EV markers (CD81, CD9, CD63, and Alix), along with many other known EV-enriched proteins, predominantly eluted in the early SEC fractions (Figure 8c,d) corresponding to the EV particles, as expected. Moreover, several proteins were identified exclusively in these early EV-containing fractions alongside the EV markers, suggesting that they are encapsulated within EVs or otherwise tightly EV associated. Conversely, some proteins traditionally regarded as EV associated (including entries from the top 100 EV marker list) were detected not only in the EV-rich early fractions but also in later fractions that lacked canonical EV markers, implying that a portion of these proteins circulates in plasma outside of EVs (Figure 8d,e), for instance, transferrin receptor (TFRC), integrin β1 (ITGB1), various heat shock proteins, and galectin-3–binding protein (LGALS3BP). PRM also confirmed that the abundant plasma proteins (e.g., IgG and albumin) predominantly appeared in later fractions. A composite purity score peaked in fraction 7 at 0.9 and fell below 0.3 after fraction 12. All together, these observations demonstrate the value of SEC fractionation for distinguishing EV-encapsulated proteins from those present as the EV-free form and highlight that analyzing specific fractions can provide complementary insights, identifying proteins uniquely co-isolated with EVs and revealing which putative proteins are also present as free-circulating entities.

## 4. Discussion

In this study, our comparative analysis revealed clear differences in EV marker recovery among the methods. These results are summarized in Table 1. The IZ SEC approach captured 62 of the top 100 EV-enriched proteins (per ExoCarta/Vesiclepedia), markedly more than any other method. The next-best approach, the T14 abundant-protein depletion kit, captured 35 of 100, whereas the polymer-based precipitation kits and the TI reagent recovered only on the order of 10–17 out of 100. Notably, several canonical exosome markers—including tetraspanins (CD9 and CD81), Alix, flotillin-1, syntenin-1, and others—were detected only in the SEC-isolated EVs and were virtually absent from the precipitated EV samples. In the precipitation and simple spin isolates, some proteins that appeared as “EV markers” were actually common plasma components (e.g., albumin, fibronectin, and complement C3). Overall, the SEC method yielded the most enriched EV proteome, with far less interference from plasma proteins, underscoring its advantage in isolating bona fide EV components. This observation is in line with prior reports that SEC-based protocols produce cleaner EV preparations and deeper proteomic coverage than traditional ultracentrifugation or PEG-based precipitation protocols. Indeed, Vanderboom et al. (2021) found that an SEC workflow identified significantly more plasma EV proteins (with higher quantitative precision) than several conventional techniques, reinforcing SEC’s status as a highly MS-compatible method [3].

Isolating EVs enabled the detection of over 150 proteins that we did not detect in whole plasma. The vast majority of these “new” proteins were discovered in the SEC (IZ) isolates (with a smaller contribution from T14 depletion), whereas the other methods yielded virtually none. This underscores that removing or bypassing abundant plasma proteins can unveil previously masked low-abundance proteins, ideal for biomarker discovery. However, because T14 is not EV specific, it recovered only about half the EV markers that SEC did and still co-purified a moderate amount of plasma proteins. Thus, protein depletion can expand the detectable proteome, but it does not enrich EVs as specifically or effectively as IZ SEC.

A key theme emerging from our results is the trade-off between EV yield and purity. The polymer-based precipitation methods (EQ, ES, and TI) and the T14 kit all yielded substantial numbers of particles, with the Total Exosome Isolation reagent in particular producing the highest particle count (approximately 50 billion particles isolated per mL of plasma, over 2.5-fold more than the others). However, this higher particle yield did not translate to a superior EV protein output for proteomics. In fact, the TI method’s proteomic profile was heavily dominated by plasma proteins (~71% overlap with the whole plasma dataset) and identified very few unique EV proteins. This illustrates that simply maximizing particle count can be misleading. Consistent with this, our purity metrics and PRM measurements showed that the TI and ES preparations had the lowest purity scores, comparable to that of whole plasma. These observations suggest that methods maximizing yield often co-isolate substantial contaminants. In our hands, the methods that returned the most particles (TI and ES) also produced proteomes very similar to whole plasma, indicating major non-EV content. By contrast, SEC yielded a somewhat lower particle count, yet those particles were highly enriched in EV markers and have reduced plasma proteins, a more compatible outcome for proteomic analysis.

One unique aspect of our study was the use of a targeted PRM mass spectrometry assay to quantitatively assess EV purity and contamination. Traditionally, EV purity is evaluated by Western blotting for a few markers or by the particle-to-protein ratio. Here, by multiplexing ~50 peptides in a PRM assay, we quantitatively measured multiple EV markers and contaminants in each sample, yielding an empirical “purity score” for each isolation. PRM was necessary for estimating EV purity. While the DDA LFQ workflow provided global proteome coverage, its data-dependent nature led to frequent missing values for low-abundance EV markers and made it difficult to use the data for purity assessment. For example, in the DDA LFQ dataset, tetraspanins such as CD81, CD9, and Alix were robustly detected in IZ samples but were often missed or completely absent (zero LFQ intensity) in EQ, ES, TI, or T14 isolates. This all-or-none detection limited our ability to calculate meaningful enrichment scores, as the scores collapsed to binary values (0 or 1). By contrast, PRM targeted these markers with higher sensitivity and reproducibility, enabling us to quantify them not only in IZ but also in other isolates (e.g., EQ and T14). This resulted in a graded range of enrichment scores (between 0 and 1) that more accurately reflected relative purity. Furthermore, PRM detected additional EV proteins (e.g., CD63 and others) that were not consistently observed in the DDA runs. The PRM results closely mirrored the untargeted proteomics: IZ had the highest purity score, whereas precipitation methods (ES and TI) had the lowest. In practical terms, the IZ preparation contained about 4–5-fold less contaminant protein than the precipitated EV samples. Thus, the targeted PRM assays provided complementary, sensitive quantification that was essential for our purity assessment and for benchmarking EV isolation methods.

SEC fractionation experiment provided further insights into the distribution of EVs and contaminants. Consistent with elution of EV particles, canonical EV markers (e.g., CD9, CD63, CD81, and Alix) were detected predominantly in the early EV-rich fractions and were largely absent from the later fractions. Interestingly, some proteins often regarded as EV associated were found not only in the EV-containing fractions but also in the EV-depleted late fractions, implying that a portion of these proteins circulates in plasma outside of EVs. For example, transferrin receptor and galectin-3–binding protein (LGALS3BP)—frequently reported in EV proteomes—appeared in both the EV-rich pools and the fractions lacking EV markers. The fractionation also confirmed that the major plasma contaminants (immunoglobulins, albumin, etc.) were confined to the late fractions. SEC fractionation not only isolates EVs with high purity but also enriches for disease-relevant proteins that would be masked in bulk plasma or co-isolated by other methods. For example, the early EV-rich fractions concentrate biomarkers such as SLC2A1, PECAM1, and APOE to levels 2–3-fold above background, revealing candidate proteins that might otherwise be undetectable. Overall, this SEC fractionation approach proved valuable for distinguishing genuine EV-encapsulated proteins from co-isolated soluble proteins, and it enabled the recovery of an EV-depleted plasma fraction that could be used for additional analyses.

Beyond our immediate experimental comparisons, having a robust quantitative purity assessment carries broader significance, especially as EV-based therapeutics move toward clinical application. EV products intended for therapeutic use must meet stringent quality and safety criteria, including the minimization of co-isolated proteins or other contaminants [15,16]. A PRM-based purity scoring assay could be readily adopted as a quality control tool in EV manufacturing, for example, to verify that a given production batch contains acceptably low levels of albumin, immunoglobulins, or other undesirable proteins. Such an approach would complement existing release tests (e.g., particle counts, sterility, and potency assays) by providing a detailed molecular purity profile. Incorporating proteomic purity metrics into Good Manufacturing Practice (GMP) workflows for EV therapeutics may facilitate regulatory approval and ensure consistent product purity, ultimately improving the safety and efficacy of EV-based therapeutic products.

Despite the strengths of our study, several limitations should be considered. We employed a bottom-up (shotgun) proteomic approach, which infers canonical proteins from peptide fragments rather than directly detecting intact proteoforms. As a result, the true complexity of proteoforms, including intact protein, splice isoforms, and post-translational modifications, may not have been fully captured. In addition, we used plasma (commercial pooled plasma) from healthy individuals and did not specifically examine variability across different donors, sexes, ages, or disease states. The performance of these isolation methods may differ with diverse sample types or clinical conditions, so caution is warranted in generalizing our findings.

It should also be noted that the total number of EV proteins identified in our study (~450 overall) is lower than some recent reports that identified >2000 EV proteins [17,18,19]. This disparity is largely due to differences in experimental design: those studies used large plasma volumes, multi-step fractionation or affinity enrichments, and merged data from multiple samples, thereby achieving greater depth. Our primary objective was to compare five single-step isolation methods under identical conditions, focusing on reproducibility and purity metrics rather than maximizing total proteome depth. We used relatively small plasma volumes (150–500 µL, e.g., 150 µL for SEC/IZ) and performed single-injection LC-MS/MS analyses with a 72 min gradient on an Orbitrap Eclipse. This design enabled consistent side-by-side evaluation but inherently limits proteome coverage compared with studies optimized for discovery. For example, Muraoka et al. [17] reported 4079 EV proteins using affinity capture combined with extensive Data-Independent Acquisition (DIA), a strategy that maximizes sampling of low-abundance peptides and minimizes missing values across runs. Similarly, Sharma et al. [18] achieved ~2000 proteins by starting with larger plasma volumes, using a different SEC format, and incorporating HiRIEF peptide prefractionation before LC-MS, substantially increasing identifications. These methodological differences utilized larger input, multi-fraction workflows, and/or DIA and naturally yield higher protein counts than our single-run DDA approach. We emphasize that while our Eclipse instrument is state of the art, label-free DDA in single runs typically identifies fewer proteins than DIA- or fractionation-based workflows. The trade-off is deliberate: our study prioritized a broad, comparative evaluation of isolation methods (five conditions × ≥ 3 replicates) over maximum depth. Importantly, our numbers are consistent with expectations for the given input and design. Notably, our findings align with expectations for single-injection plasma EV proteomics; even state-of-the-art instruments typically detect ~700–1000 proteins in unfractionated plasma [20], owing to the dominance of a few proteins. The incremental proteins gained by the best methods (SEC and T14) in our study, with roughly +50–80 proteins over 240 found in whole plasma (Figure 2c), represent biologically meaningful, low-abundance EV components unveiled by removing high-abundance proteins. Furthermore, we evaluated each isolation technique in its standard one-step format and did not explore combined or sequential workflows. Multi-step approaches (for example, SEC followed by immunoprecipitation) may achieve cell-specific EV purity. Overall, our comparative findings offer practical guidance for selecting effective EV isolation methods and underscore the importance of rigorous purity assessments in plasma EV proteomics.

## 5. Conclusions

Our comprehensive comparison of plasma EV isolation methods shows that SEC (qEV column) produces EV samples of markedly higher purity and proteomic richness than the other evaluated techniques. The SEC-isolated EV proteome was the most distinct from plasma, containing many more recognized EV markers and novel proteins and far fewer contaminating proteins. Precipitation-based methods and simple protein depletion, while easier or yielding more particles, were inferior in terms of EV specificity and introduced considerable plasma protein carryover. These results agree with a body of emerging literature pointing to SEC (and related chromatographic or affinity methods) as optimal for EV proteomics when the goal is to discover or quantify low-abundance vesicle proteins. We also demonstrate the utility of a PRM-based quantitative purity assessment for EV isolates, which could help standardize method evaluations across different studies. Moving forward, the reliability of EV-based biomarker discovery can be enhanced by (for example) implementing standardized isolation protocols and including quantitative purity assessments for each preparation. By rigorously confirming that an EV sample is free of excess contaminants through multi-marker PRM panels or similar assays, researchers can ensure that observed disease biomarkers truly originate from EVs. Such practices will improve reproducibility and accelerate the translation of circulating EVs into clinical diagnostics.

## Figures and Tables

**Table 1:** Comparison of plasma EV isolation methods tested, with references and key findings from this study.

Method	Principle/Reference	Key Findings in This Study
ExoQuick (EQ)	Polymer-based precipitation of EVs; manufacturer’s protocol (System Biosciences) [2].	High particle yield (~6–20 × 10^10^/mL plasma), but ~20% proteome overlap with plasma; modest enrichment of EV markers (17/100 ExoCarta/Vesiclepedia); significant co-isolation of plasma proteins; moderate purity score.
ExoSpin (ES)	Precipitation followed by column purification (Cell Guidance Systems) [2].	Particle yield similar to EQ, but high proteome overlap with plasma (~73%); very low EV marker recovery (10/100); poor depletion of albumin/IgG (~60–70% removed only); lowest composite purity score (≈0.26).
SEC (IZ)	Size-exclusion chromatography using Izon qEV 35 nm columns [3].	Highest EV marker recovery (62/100), including tetraspanins CD9, CD81, CD63, and ALIX; strong depletion of albumin/IgG (>90%); lowest plasma proteome overlap (~11%); 61 unique proteins detected absent in plasma; best purity composite score (~0.83); also enabled separate recovery of EV-depleted plasma fractions.
Total Exosome Isolation (TI)	Polymer-based precipitation reagent (ThermoFisher Scientific).	Highest particle yield (>50 × 10^10^/mL plasma; ~2.5× other methods), but ~71% proteome overlap with plasma; very poor EV marker recovery (15/100); identified few unique proteins (<5); composite purity score is low (~0.28).
Top14 depletion (T14)	Immuno-depletion of 14 most abundant plasma proteins (ThermoFisher kit; targets include albumin, immunoglobulins (IgA, IgD, IgE, IgG, and IgM), transferrin, haptoglobin, fibrinogen, α1-acid glycoprotein, α1-antitrypsin, α2-macroglobulin, and apolipoprotein A-I/A-II).	Removed majority of Top14 proteins (~95% of plasma mass); recovered 35/100 EV markers; identified 54 unique proteins not seen in plasma (2nd highest after SEC); however, not EV specific, and moderate contamination persisted; purity score intermediate (~0.50).

## Data Availability

The raw data supporting the conclusions of this article will be made available by the authors on request.

## Supplementary Materials

The following supporting information can be downloaded at: https://www.mdpi.com/article/10.3390/proteomes13030045/s1: Table S1. Particle count and protein content of the isolated EVs; Table S2. Relative levels of the most frequently observed EV markers detected in the 5 EV preparations (EQ, ES, IZ, TI, and T14) and starting plasma PL; Table S3. Relative levels plasma proteins detected in the 5 EV preparations (EQ, ES, IZ, TI, and T14) and starting plasma PL; Table S4. Relative levels proteins detected in the 5 EV preparations (EQ, ES, IZ, TI, and T14) but absent in starting plasma PL; Table S5. Peptides used for PRM analysis; Table S6. List of positive and negative markers used for purity assessment; Figure S1. Original uncropped version of the image shown in Figure 8b of the Coomassie blue-stained SDS-PAGE of pooled IZ fractions and starting plasma. The lanes correspond to (left to right): protein molecular weight markers; pooled IZ fractions 6–8, 9–10, 11–12, 13–14, 15–16, 17–18, and 19–20; blank lane; and starting plasma.
