# Supplementary figures and images for: Comparative Analysis of Plasma Extracellular Vesicle Isolation Methods for Purity Assessment and Biomarker Discovery
Source: Proteomes. 2025 Sep 18;13(3):45. doi: 10.3390/proteomes13030045 (PMC12452325; doi:10.3390/proteomes13030045)

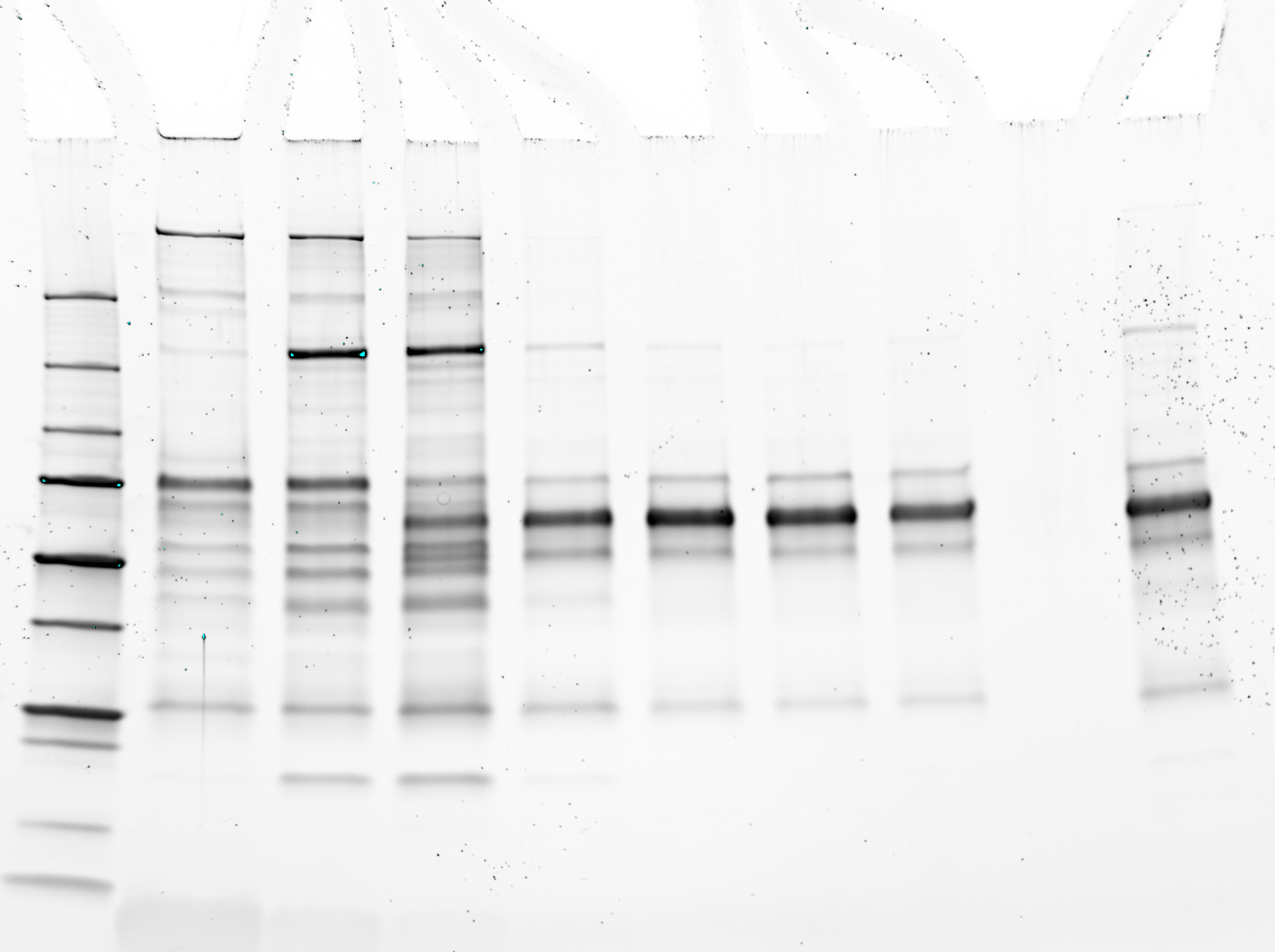

Supplement: Supplementary file 1 [file proteomes-13-00045-s001.zip › proteomes-3823593-Figure S1.tif]
